# Expressed Emotions in Families of Patients With Bipolar Disorder

**DOI:** 10.7759/cureus.82847

**Published:** 2025-04-23

**Authors:** Minakshi Verma, Koustubh R Bagul, Riya Gangwal, Pali Rastogi, Varchasvi Mudgal, Akanksha Singh

**Affiliations:** 1 Psychiatry, Mahatma Gandhi Memorial (MGM) Medical College, Indore, IND

**Keywords:** bipolar disorder, caregiver burden, expressed emotion, family dynamics, mental health caregiving

## Abstract

Introduction

Bipolar affective disorder (BPAD) is a chronic psychiatric illness characterized by recurrent episodes of mania, hypomania, and depression, significantly impacting both patients and their caregivers. Expressed emotion (EE), which includes criticism, hostility, and emotional over-involvement, plays a crucial role in caregiver burden and patient outcomes. High EE environments have been linked to increased relapse rates and hospitalization, while low EE settings promote better adherence and stability. In India, where mental healthcare is predominantly family-driven, caregivers experience significant emotional, social, and financial strain. This study evaluates EE among caregivers of BPAD patients in an Indian setting and examines its association with patient and caregiver characteristics.

Methods

A cross-sectional study was conducted among 120 primary caregivers of BPAD patients attending a tertiary care psychiatry outpatient department in Central India. Participants were selected based on specific inclusion and exclusion criteria. The Family Attitude Scale (FAS) was used to assess expressed emotions, while socio-demographic and clinical variables were recorded. Pearson’s correlation coefficient was applied to examine associations between EE and patient characteristics. Data were analyzed using SPSS version 26 (IBM Corp., Armonk, NY, USA), with statistical significance set at p < 0.05.

Results

Caregivers were predominantly male (72, 60%), married (116, 96.7%), and had an average age of 40.76 ± 13.9 years. The mean FAS score was 67.68 ± 7.4, indicating a high level of expressed emotions. FAS scores showed a significant positive correlation with caregiver age (r = 0.404, p < 0.001) and years of cohabitation (r = 0.239, p = 0.008). Among patients, higher FAS scores were associated with increased illness duration (r = 0.601, p < 0.001), number of hospitalizations (r = 0.433, p < 0.001), and time spent in the hospital (r = 0.306, p = 0.002). These findings indicate that prolonged caregiving and severe patient illness contribute to heightened EE levels.

Conclusion

Caregivers of BPAD patients in India experience high levels of EE, influenced by demographic factors and the severity of the patient’s illness. High levels of expressed emotion observed among caregivers highlight the importance of targeted psychoeducation and family-focused interventions aimed at reducing EE. Addressing EE within families may contribute to improved illness course and reduced relapse risk in patients with bipolar disorder. Future research should explore culturally appropriate strategies to manage EE and strengthen supportive family environments.

## Introduction

Bipolar affective disorder (BPAD) is a long-term mental health condition characterized by repeated episodes of mania, hypomania, and depression, impacting both patients and their caregivers [1]. It is one of the leading causes of disability worldwide, affecting around 1% of the global population [2]. Caregivers play a crucial role in managing symptoms, ensuring medication adherence, and maintaining continuity of care for individuals with BPAD [3].

Caring for someone with BPAD can be emotionally, financially, socially, and physically exhausting [4]. The unpredictable nature of the illness increases stress for caregivers, as they navigate sudden mood shifts and crises [5]. One major factor affecting their well-being is expressed emotion (EE), which includes hostility, criticism, and emotional over-involvement in family interactions [6]. Studies have shown that high EE is linked to higher relapse rates, poor treatment adherence, and increased hospitalization [7]. On the other hand, a supportive, low-EE environment helps patients maintain stability, follow treatment plans, and experience better overall outcomes [8].

Most studies on caregiver experiences and family involvement in BPAD have been conducted in Western countries, with limited data from India [9]. In India, mental health care primarily falls on families, with caregivers expected to provide unconditional support despite the toll it takes on their well-being [10]. Unlike in high-income countries, professional caregiving services are scarce, meaning family members shoulder most of the responsibility [11]. Caregivers also face financial difficulties, social stigma, and a lack of mental health resources, all of which contribute to heightened EE and added stress [12].

This study aims to assess EE among caregivers of BPAD patients in India, examine its relationship with patient and caregiver characteristics, and explore culturally appropriate interventions to reduce stress and improve outcomes for both caregivers and patients.

## Materials and methods

This cross-sectional study was conducted among primary caregivers of patients diagnosed with BPAD attending the Psychiatry Outpatient Department at a tertiary care hospital in Central India. Patients were diagnosed based on ICD-10 criteria.

Caregivers aged 18 years or older, living with the patient for at least one year, and actively involved in caregiving without receiving wages were included. Caregivers with organic or psychiatric disorders affecting cognitive functioning or those unwilling to participate were excluded.

The sample size was calculated using the prevalence formula

$n=\frac{z^{2}p(1-p)}{d^{2}}$​​​​​​

where Z = 1.96 (95% confidence level), P = 0.8 (80% hospital-based prevalence of caregiver burden from Indian studies), and d = 0.07 (margin of error). The sample size was calculated to be 125, but 120 caregivers were included for feasibility while maintaining statistical power.

Participants were recruited by convenience sampling from the psychiatry outpatient department.

The study was conducted over six months, from February to July 2024. Data were collected using the Family Attitude Scale (FAS) [13], a 30-item self-report measure that assesses expressed emotions such as criticism and hostility. Each item is scored from 0 to 4, yielding a total score ranging from 0 (low EE) to 120 (high EE), with higher scores indicating greater levels of expressed emotion.

Data were analyzed using SPSS version 26 (IBM Corp., Armonk, NY, USA). Descriptive statistics summarized demographic characteristics, and Pearson’s correlation coefficient examined relationships between expressed emotions and caregiving-related factors, with statistical significance set at p < 0.05.

Ethical clearance was obtained from the Institutional Ethics Committee of Mahatma Gandhi Memorial (MGM) Medical College, Indore, under letter number EC/MGM/Feb-24/30 dated 17th February 2024. Written informed consent was obtained from all participants, and confidentiality was maintained throughout the study.

## Results

The study primarily involved 85 (70.8%) male patients, with the majority being married (70, 58.3%) and having completed secondary education (52, 43.3%) (Table 1). Among the caregivers, most were aged 21-40 years, predominantly male (72, 60.0%), and married (116, 96.7%). In terms of education, 47 (39.2%) had secondary education, and 24 (20.0%) had no formal education. Caregivers were mostly from the lower middle class (53, 44.2%) and held roles as unskilled workers or in clerical/shop owner/farmer positions (33, 27.5% each). Spouses (41, 34.2%) and fathers (28, 23.3%) were the primary caregivers.

**Table 1:** Socio-Demographic data of caregivers and patients

S.No	Variables	Caregivers		Patients	
		N=120	%	N=120	%
Age in years	<20	2	1.7	2	1.7
	21-30	36	30	54	45
	31-40	29	24.2	48	40
	41-50	14	11.7	10	8.3
	51-60	27	22.5	6	5
Gender	Male	72	60	85	70.8
	Female	48	40	35	29.2
Marital Status	Married	116	96.7	70	58.3
	Unmarried	4	3.3	48	40
	Separated	-	-	1	0.8
	Divorced	-	-	1	0.8
Education	No formal education	24	20	10	8.3
	Primary	19	15.8	19	15.8
	Secondary	47	39.2	52	43.3
	Higher secondary	22	18.3	-	-
	Graduate	8	6.7	22	18.3
Occupation	Unemployed	31	25.8	-	-
	Unskilled	33	27.5	-	-
. -	Semi-Skilled	10	8.3	-	-
	Skilled	13	10.8	-	-
.	Clerical/Shop owner/Farmer	33	27.5	-	-
Socio-Economic Status.	Upper	0	0	-	-
.	Upper middle	17	14.2	-	-
	Lower middle	53	44.2	-	-
	Upper Lower	16	13.3	-	-
	Lower	34	28.3	-	-
Relationship with patient	Father	28	23.3	-	-
.	Mother	20	16.7	-	-
	Spouse	41	34.2	-	-
	Others (siblings, in-laws)	31	25.8	-	-

Caregivers had an average age of 40.76 ± 13.9 years, had lived with the patient for an average of 17.43 ± 9 years, and spent 8.78 ± 2.9 hours per day with the patient. The mean FAS score was 67.68 ± 7.4 (on a scale of 0 to 120), suggesting elevated levels of expressed emotions (Table 2). 

**Table 2:** Characteristics of caregivers

Variable	Mean	Standard deviation	Range (min-max)
Age	40.76	13.974	19-70
Time spent with patient in same house (years)	17.43	9.079	2yrs-48yrs
Time spent with patient during day (hours)	8.78	2.920	2 hrs-18 hrs
Family Attitude Scale (FAS)	67.68	7.496	53-78

Pearson correlation analysis showed a moderate positive correlation of FAS with caregiver age (r = 0.404, p < 0.001) and a modest correlation with years living with the patient (r = 0.239, p = 0.008). There was no significant link with daily caregiving hours (r = -0.006, p = 0.951) (Table 3).

**Table 3:** Correlation of Family Attitude Scale (FAS) with caregiver characteristics

	Correlation coefficient	P value
FAS and age of caregiver	0.404	0.000
FAS and time spent with patient in same house (years)	0.239	0.008
FAS and time spent with patient during day (hours)	-0.006	0.951

The average duration of illness was 5.59 ± 3.78 years, with patients having an average of 1.76 ± 1.73 hospitalizations, each lasting an average of 12.78 ± 9.87 days. On average, patients experienced 2.63 ± 2.21 manic episodes, 0.49 ± 0.65 depressive episodes, and infrequent suicide or homicide attempts (Table 4).

**Table 4:** Characteristics of patients

Variable	Mean	Standard deviation	Range
Total duration of illness	5.59	3.78	0.04-25
Number of hospitalisation	1.76	1.734	0-8
Time spent in hospital (days)	12.78	9.867	0-48
Number of episodes of mania	2.63	2.211	1-16
Number of episodes of depression	0.49	0.648	0-3
Number of attempt of suicide	0.12	0.322	0-1
Number of attempt of homicide	0.04	0.24	0-2

FAS scores were significantly associated with patient age (r = 0.404, p < 0.001), illness duration (r = 0.601, p < 0.001), hospitalizations (r = 0.433, p < 0.001), and hospitalization days (r = 0.306, p = 0.002) (Table 5).

**Table 5:** Correlation of Family Attitude Scale (FAS) with patient characteristics

	Correlation coefficient	P value
FAS and age of patient	0.404	0.000
FAS and total duration of illness	0.601	0.001
FAS and number of hospitalisations	0.433	0.001
FAS and time spent in hospital (days)	0.306	0.002

## Discussion

This study highlights the substantial burden experienced by caregivers of BPAD patients, particularly in terms of EE. Our findings align with prior research, showing that high EE is associated with worse patient outcomes, as evidenced by more severe and prolonged illness, a greater number of hospitalizations, and longer durations of hospital stay [6,14]. Studies suggest that hostility, critical attitudes, and excessive emotional involvement contribute to poor treatment adherence and frequent relapses in BPAD [7,15].

Indian caregivers often belong to joint family systems, where caregiving duties are shared across multiple family members [16]. However, primary caregivers bear the bulk of responsibilities, leading to increased emotional distress and high EE [10]. Financial constraints, social stigma, and lack of structured support services exacerbate EE among Indian caregivers, aligning with findings from previous studies [17]. Unlike in Western countries, where formal caregiving institutions and respite care exist, Indian caregivers must manage BPAD symptoms within the household, heightening stress levels [11,18]. Caregivers from lower socioeconomic backgrounds are particularly vulnerable, as financial struggles limit access to psychiatric care and psychoeducation programs [12,19].

This study also establishes a strong link between patient characteristics and caregiver EE. Caregivers of patients with longer illness duration, more hospitalizations, and severe manic episodes reported significantly higher EE levels, likely due to the cumulative stress of prolonged caregiving [20]. Previous studies confirm that as illness severity increases, caregiver burden rises, leading to higher EE and emotional exhaustion [21]. Perlick et al. (2007) similarly observed that caregiver stress increases over time, creating a feedback loop where high EE contributes to poor patient outcomes, which in turn heightens caregiver burden [11,22].

Addressing EE in caregivers is essential for improving outcomes for both patients and families. Psychoeducation on its own has been shown to reduce caregiver distress, improve family communication, and enhance treatment adherence [23]. To extend these benefits, structured EE-reduction workshops - brief, skills-based group sessions modelled on family psycho-educational programmes - can be incorporated into routine follow-up clinics [22]. Parallel mindfulness-based caregiver training lowers perceived stress and emotional reactivity, thereby indirectly decreasing EE levels [10]. Community-based caregiver support groups can reinforce these formal interventions by fostering peer networks within primary health centre settings [24]. Integrating psychoeducation, mindfulness modules, and EE-focused communication drills into standard family therapy may further reduce distress and strengthen family dynamics [25]. Finally, policy actions such as targeted financial support, caregiver stipends, and the respite-care services advocated in the WHO Mental Health Action Plan [26] could buffer the long-term burden that perpetuates high EE.

A key strength of this study is the use of a validated Hindi version of the Family Attitude Scale in a relatively large, naturalistic clinic sample, allowing for reliable assessment of EE in an Indian context. By examining both caregiver and patient variables, the study adds valuable data to the limited Indian literature on EE in bipolar disorder. The finding that higher EE correlates with longer illness duration and more hospitalizations highlights the clinical need to address family emotional climate. Incorporating EE-based psychoeducation and support strategies into routine care may improve patient outcomes and caregiver well-being.

The limitations of this study include its cross-sectional design, which restricts the ability to establish causal relationships between expressed emotions and caregiver burden. Self-reported measures introduce potential biases, such as social desirability bias, affecting the accuracy of responses. Since the study was conducted at a single tertiary care hospital in Central India, its findings may not be generalizable to caregivers from diverse cultural and socioeconomic backgrounds. Additionally, the exclusion of caregivers with psychiatric or cognitive impairments may overlook an important subset experiencing significant burden. The study lacks a comparison group, which limits the ability to differentiate the burden of BPAD caregivers from those caring for individuals with other psychiatric disorders like schizophrenia. Cultural and social influences on caregiving stress are acknowledged but not deeply explored, and the study does not extensively analyze the financial and gender-based aspects of caregiver burden. Furthermore, the absence of objective clinical measures and reliance on patient-reported data may affect the robustness of findings.

Future directions include longitudinal follow‑up and controlled trials of family‑based interventions (e.g., structured psychoeducation or EE‑reduction workshops) to determine whether lowering EE shortens episodes and reduces rehospitalisation.

## Conclusions

Caring for someone with bipolar disorder can be overwhelming, especially when emotional strain and financial stress pile up. High levels of EE were common in our sample and were correlated with longer illness duration and more frequent hospitalisations rather than treatment-related “poor outcomes” per se. With limited formal caregiving support in India, families are left to manage on their own. Targeted measures like psychoeducation, brief EE-reduction workshops, mindfulness-based caregiver programmes, and community peer groups offer practical ways to lower EE and strengthen family relationships. Integrating these efforts into routine psychiatric care, along with financial and policy support, can make a real difference in both caregiver well-being and patient recovery.

## Appendices

**Table 6:** Family Attitude Scale (FAS-30) Reproduced from Kavanagh DJ, O’Halloran P, Manicavasagar V, Clark D, Piatkowska O, Tennant C, Rosen A. The Family Attitude Scale: reliability and validity of a new scale for measuring the emotional climate of families. Psychiatry Research. 1997; 70, 185–195 [13], with permission from the original authors and publisher.

S. No.	Statement	Every day	Most days	Some days	Very rarely	Never
1	It is good to have him/her around	☐	☐	☐	☐	☐
2	He/she makes me feel drained	☐	☐	☐	☐	☐
3	He/she ignores my advice	☐	☐	☐	☐	☐
4	He/she is really hard to take	☐	☐	☐	☐	☐
5	I shout at him/her	☐	☐	☐	☐	☐
6	I wish he/she were not here	☐	☐	☐	☐	☐
7	I feel that he/she is driving me crazy	☐	☐	☐	☐	☐
8	I lose my temper with him/her	☐	☐	☐	☐	☐
9	He/she is easy to get along with	☐	☐	☐	☐	☐
10	I am sick of having to look after him/her	☐	☐	☐	☐	☐
11	He/she deliberately causes me problems	☐	☐	☐	☐	☐
12	I enjoy being with him/her	☐	☐	☐	☐	☐
13	He/she is a real burden	☐	☐	☐	☐	☐
14	I argue with him/her	☐	☐	☐	☐	☐
15	I feel very close to him/her	☐	☐	☐	☐	☐
16	I can cope with him/her	☐	☐	☐	☐	☐
17	Living with him/her is too much for me	☐	☐	☐	☐	☐
18	He/she is infuriating	☐	☐	☐	☐	☐
19	I find myself saying nasty or sarcastic things to him/her	☐	☐	☐	☐	☐
20	He/she appreciates what I do for him/her	☐	☐	☐	☐	☐
21	I feel that he/she is becoming easier to live with	☐	☐	☐	☐	☐
22	I wish he/she would leave me alone	☐	☐	☐	☐	☐
23	He/she takes me for granted	☐	☐	☐	☐	☐
24	He/she can control himself/herself	☐	☐	☐	☐	☐
25	He/she is hard to get close to	☐	☐	☐	☐	☐
26	I feel that he/she is becoming harder to live with	☐	☐	☐	☐	☐
27	I feel very frustrated with him/her	☐	☐	☐	☐	☐
28	He/she makes a lot of sense	☐	☐	☐	☐	☐
29	I feel disappointed with him/her	☐	☐	☐	☐	☐
30	He/she tries to get along with me	☐	☐	☐	☐	☐
